# GLMdenoise: a fast, automated technique for denoising task-based fMRI data

**DOI:** 10.3389/fnins.2013.00247

**Published:** 2013-12-17

**Authors:** Kendrick N. Kay, Ariel Rokem, Jonathan Winawer, Robert F. Dougherty, Brian A. Wandell

**Affiliations:** ^1^Department of Psychology, Washington University in St. LouisSt. Louis, MO, USA; ^2^Department of Psychology, Stanford UniversityStanford, CA, USA; ^3^Department of Psychology, New York UniversityNew York, NY, USA; ^4^Center for Cognitive and Neurobiological Imaging, Stanford UniversityStanford, CA, USA

**Keywords:** BOLD fMRI, general linear model, cross-validation, signal-to-noise ratio, physiological noise, correlated noise, ICA, RETROICOR

## Abstract

In task-based functional magnetic resonance imaging (fMRI), researchers seek to measure fMRI signals related to a given task or condition. In many circumstances, measuring this signal of interest is limited by noise. In this study, we present *GLMdenoise*, a technique that improves signal-to-noise ratio (SNR) by entering noise regressors into a general linear model (GLM) analysis of fMRI data. The noise regressors are derived by conducting an initial model fit to determine voxels unrelated to the experimental paradigm, performing principal components analysis (PCA) on the time-series of these voxels, and using cross-validation to select the optimal number of principal components to use as noise regressors. Due to the use of data resampling, GLMdenoise requires and is best suited for datasets involving multiple runs (where conditions repeat across runs). We show that GLMdenoise consistently improves cross-validation accuracy of GLM estimates on a variety of event-related experimental datasets and is accompanied by substantial gains in SNR. To promote practical application of methods, we provide MATLAB code implementing GLMdenoise. Furthermore, to help compare GLMdenoise to other denoising methods, we present the *Denoise Benchmark (DNB)*, a public database and architecture for evaluating denoising methods. The DNB consists of the datasets described in this paper, a code framework that enables automatic evaluation of a denoising method, and implementations of several denoising methods, including GLMdenoise, the use of motion parameters as noise regressors, ICA-based denoising, and RETROICOR/RVHRCOR. Using the DNB, we find that GLMdenoise performs best out of all of the denoising methods we tested.

## Introduction

The blood oxygenation level dependent (BOLD) signal measured in functional magnetic resonance imaging (fMRI) arises from multiple sources. The portion of the BOLD signal arising from neural activity is generally of scientific interest. Other portions of the BOLD signal reflect various physiological and instrumental factors, and these are typically unwanted and considered to be noise. Being able to separate signal from noise has clear value for scientific experiments.

In task-based fMRI, researchers seek to identify signals that are related to an experimental manipulation, such as a sensory stimulus, motor act, or cognitive process. This is challenging due to the presence of many sources of noise (e.g., physiological noise, instrumental noise) in the BOLD signal. To improve sensitivity to task-related signals, a simple and effective approach is to use block experimental designs (Liu et al., 2001). In block designs, experimental conditions have long durations (e.g., 12 s). This elicits (or is likely to elicit) sustained neural activity and leads to a large BOLD response. However, in many circumstances, block designs conflict with the experimental goals, and researchers must use event-related designs where conditions are brief (e.g., 1 s). For example, event-related designs may be necessary to avoid adaptation and anticipatory effects (Zarahn et al., 1997; Josephs and Henson, 1999), to sample many conditions (Kay et al., 2008b; Kriegeskorte et al., 2008), to examine the temporal dynamics of the BOLD response to a single event (Ploran et al., 2007; Ho et al., 2009; Huettel, 2012), or to match the duration of the stimulus to a psychophysical threshold (Grill-Spector and Kanwisher, 2005).

An alternative approach for improving sensitivity is to incorporate nuisance regressors into a general linear model (GLM) analysis of fMRI data (Friston et al., 1995; Lund et al., 2006). In this approach, a linear model is specified that includes not only task-related regressors describing the effects of experimental events but also nuisance regressors describing likely sources of noise. If the nuisance regressors successfully capture some of the noise, then this may improve estimates of the task-related components of the BOLD signal. However, denoising via nuisance regressors depends critically on the selection of regressors: if the regressors are inaccurate or fail to capture a significant portion of the noise, they may have little effect or even worsen task-related estimates.

Auxiliary physiological measurements can be useful for constructing nuisance regressors. The RETROICOR method (Glover et al., 2000) and variations thereof (e.g., Birn et al., 2006; Shmueli et al., 2007; Chang et al., 2009; Hagberg et al., 2012) use cardiac and respiratory measurements to predict physiological effects in the BOLD signal, and these can be used as nuisance regressors. However, a limitation of these methods is that their effectiveness depends on stable and accurate physiological measurements and an accurate model of how physiological processes relate to the BOLD signal. Furthermore, these methods can capture only some sources of noise in the data.

Recently, Bianciardi et al. (2009b) described a method for estimating nuisance regressors directly from BOLD data (see also Fox et al., 2006; Behzadi et al., 2007). In their method, a region-of-interest activated by a task is identified in an fMRI dataset. Next, auxiliary fMRI data are collected without a task and a set of voxels whose time-series correlate with the time-series of the region-of-interest is selected. Finally, signals from the selected voxels in the original task-based experiment are used to derive nuisance regressors. These nuisance regressors may capture unwanted BOLD effects related to physiological processes (Bianciardi et al., 2009a). They may also capture correlated fluctuations in neural activity (Fox and Raichle, 2007) as well as motion effects that remain even after applying motion compensation algorithms (Lund et al., 2006).

Here, we simplify and extend the technique introduced by Bianciardi et al. (Bianciardi et al., 2009b). We describe a new technique, GLMdenoise, that requires no auxiliary fMRI data, automatically derives nuisance regressors, and automatically determines the optimal number of regressors. We demonstrate GLMdenoise on 21 experimental datasets involving a variety of event-related designs. Accurate estimation of BOLD responses in these datasets is challenging as the experiments involve a large number of conditions (between 9 and 156) that are relatively short in duration (between 1 and 5 s).

A denoising technique should produce estimates of task-related BOLD responses that accurately generalize to novel measurements; hence, we use cross-validation to evaluate the effectiveness of GLMdenoise (Kay et al., 2008a). We find that the method consistently improves cross-validation accuracy of BOLD response estimates compared to a standard GLM analysis. Furthermore, GLMdenoise yields substantial improvements in SNR, which we quantify as the maximum response amplitude observed for a voxel, divided by the error (uncertainty) on this amplitude.

We believe in the importance of developing methods that are precisely described and readily applicable to actual studies (Poline and Poldrack, 2012). In line with these values, we make available MATLAB code implementing GLMdenoise at http://kendrickkay.net/GLMdenoise/. The code takes a design matrix and fMRI time-series and returns estimates of the hemodynamic response function (HRF) and BOLD response amplitudes (beta weights). The code also returns the original time-series with nuisance components removed; this allows the code to be incorporated into existing analysis workflows (i.e., the user can choose to ignore the GLM estimates and treat the code as a pre-processing step prior to subsequent data analysis). Since the fitting process consists of large-scale matrix operations applied to many voxels simultaneously, the code is memory-intensive but fast (an entire dataset can be processed in less than 15 min).

Finally, to facilitate comparison of GLMdenoise to other denoising methods, we present the Denoise Benchmark (DNB). The DNB, available at http://kendrickkay.net/DNB/, is a public database and architecture for comparing denoising methods. The premise behind the DNB is to provide an application programming interface (API) for denoising methods; when this API is satisfied, the accuracy of a denoising method is evaluated using an automatic cross-validation procedure. The DNB consists of the datasets described in this paper, code that performs the cross-validation procedure, and implementations of several denoising methods. Using the DNB, we find that GLMdenoise outperforms a number of other denoising techniques, including the use of motion parameters as noise regressors, ICA-based denoising, and RETROICOR/RVHRCOR.

## Materials and methods

### Subjects and datasets

We collected 21 datasets from 12 experienced fMRI subjects (8 males). Informed written consent was obtained from all subjects, and the Stanford University Institutional Review Board approved the experimental protocol. Each dataset consisted of one scan session, and each scan session consisted of multiple runs, each typically lasting about 5 min.

Functional MRI data were collected at the Lucas Center at Stanford University and the Stanford Center for Cognitive and Neurobiological Imaging (CNI) using 3T MR scanners and T2^*^-weighted, single-shot, gradient-echo spiral-trajectory (Lucas Center) and echo-planar imaging (CNI) pulse sequences. Experiments involved presentation of visual stimuli while BOLD responses were measured in visual cortex. Subjects maintained central fixation on a small target throughout the experiments. In some datasets (datasets 14–21; 8 out of 21 datasets), physiological data were recorded using a pulse oximeter and a respiratory belt attached to the subject.

All experiments used an event-related design (Liu, 2012). However, designs varied substantially across experiments. The number of conditions varied between 9 and 156; the duration of each condition varied between 1 and 5 s; and the number of repetitions of each condition varied between 3 and 30. (For example, one condition in an experiment might be the presentation of a flickering checkerboard at a certain location in the visual field for 3 s, and this condition might occur 5 times over the course of the experiment). Conditions were presented in random order within each run, and rest periods were included between conditions and at the beginning and end of each run. In some datasets (datasets 10–11, 14–17, 20–21; 8 out of 21 datasets), every condition was presented at least once during a run. In other datasets (datasets 1–9, 12–13, 18–19; 13 out of 21 datasets), conditions were split across multiple runs. For example, datasets 7 and 8 involved 104 conditions which were split across two runs, each containing 52 conditions; together, the two runs comprise a run set and multiple run sets were collected over the course of the scan session. The specific characteristics of each dataset are detailed in Tables 1, 2.

**Table 1:** **Summary of datasets**.

**Dataset**	**Experiment**	**Subject**	**Number of conditions**	**Condition duration (seconds)**	**Number of repetitions per run (or run set)**	**Number of runs (or run sets)**	**Scanner**	**RF coil**	**Voxel size (mm)**	**TR (seconds)**	**TE (ms)**	**Flip angle (deg)**	**Volume dimensions**	**Number of volumes per run**	**Total number of runs**	**Physiological data collected?**
1	A	S1	69	3	1	5	3T1	N8	2.5	1.323751	29.7	71	64 × 64 × 21	270	10	No
2	A	S2	69	3	1	5	3T1	N2	2.5	1.323751	29.7	71	64 × 64 × 21	270	10	No
3	A	S3	69	3	1	5	3T1	N2	2.5	1.323751	29.7	71	64 × 64 × 21	270	10	No
4	B	S3	156	3	1	3	CNI	N32	2.5	1.337702	28	68	64 × 64 × 22	300	12	No
5	B	S2	156	3	1	3	CNI	N32	2.5	1.337702	28	68	64 × 64 × 22	300	12	No
6	B	S4	156	3	1	3	CNI	N32	2.5	1.337702	28	68	64 × 64 × 22	300	12	No
7	C	S5	104	3.5	1	7	CNI	N32	2	1.985626	31	77	80 × 80 × 26	140	14	No
8	C	S4	104	3.5	1	7	CNI	N32	2	1.985626	31	77	80 × 80 × 26	140	14	No
9	D	S2	69	3	1	5	CNI	N32	1.8	1.605242	35	73	70 × 70 × 20	225	10	No
10	E	S4	35	3	1	10	CNI	N32	2.5	1.337702	28	68	64 × 64 × 22	270	10	No
11	F	S3	9	3	2	15	CNI	N32	2.5	1.337702	28	68	64 × 64 × 22	150	15	No
12	G	S6	69	1	2	6	3T2	N8	2.5	1.323751	28.7	77	64 × 64 × 20	252	12	No
13	G	S7	69	1	2	6	3T2	N8	2.5	1.323751	28.7	77	64 × 64 × 20	252	12	No
14	H	S2	20	3	1	4	CNI	N32	2.5	1.337702	28	68	64 × 64 × 22	162	4	Yes
15	H	S3	20	3	1	4	CNI	N32	2.5	1.337702	28	68	64 × 64 × 22	162	4	Yes
16	I	S8	9	3	2	4	CNI	N32	2.5	1.337702	28	68	64 × 64 × 22	150	4	Yes
17	I	S9	9	3	2	4	CNI	N32	2.5	1.337702	28	68	64 × 64 × 22	150	4	Yes
18	J	S4	81	5	1	6	CNI	N16	2	2.006553	31	77	80 × 80 × 26	156	12	Yes
19	J	S10	81	5	1	6	CNI	N16	2	2.006553	31	77	80 × 80 × 26	156	12	Yes
20	K	S11	20	3	1	4	CNI	N32	2	2.006553	31	77	80 × 80 × 26	108	4	Yes
21	K	S12	20	3	1	4	CNI	N32	2	2.006553	31	77	80 × 80 × 26	108	4	Yes

3T1, Lucas Center at Stanford University, 3T GE Signa HDX scanner; 3T2, Lucas Center at Stanford University, 3T GE Signa MR750 scanner; CNI, Stanford Center for Neurobiological Imaging, 3T GE Signa MR750 scanner; N2, Nova quadrature RF coil; N8, Nova 8-channel RF coil; N16, Nova 16-channel visual array RF coil; N32, Nova 32-channel RF coil.

Additional notes: All datasets used a T2^*^-weighted, single-shot, gradient-echo pulse sequence, either spiral-trajectory (3T1, 3T2) or echo-planar imaging (CNI). Experiment I was identical to experiment F except that physiological data were collected and fewer runs were collected. Experiment K was identical to experiment H except for differences in pulse sequence parameters (voxel size, TR, TE, flip angle).

**Table 2:** **Details of the stimuli used in the experiments**.

**Experiment**	**Stimulus details**
A	High-contrast black-and-white noise patterns; 10 frames/s for 3 s; conditions vary with respect to the visual field location of the patterns
B	Band-pass filtered grayscale images; 3 frames/s for 3 s; conditions vary with respect to visual dimensions such as location, contrast, and orientation
C	Arrays of grayscale faces and hands; 2 frames/s for 3.5 s; conditions vary with respect to whether faces or hands composed the arrays and with respect to the spatial layout of the arrays
D	Color textures composed of letters of different colors and sizes; 3 frames/s for 3 s; conditions vary with respect to the visual field location of the textures
E	Band-pass filtered grayscale objects; 3 frames/s for 3 s; each condition involves flashed presentation of one distinct object
F	High-contrast black-and-white noise patterns; 3 frames/s for 3 s; conditions vary with respect to the type and visual field location of the patterns
G	Achromatic white noise; 10 frames/s for 1 s; conditions vary with respect to the visual field location of the noise
H	Same as E
I	Same as F
J	Grayscale faces; 4 frames/s for 5 s; conditions vary with respect to the visual field location of the faces
K	Same as E

### Data pre-processing

The GLMdenoise technique is designed to be applied to fMRI data following general pre-processing steps. For the datasets in this paper, we performed the following pre-processing steps: the first five volumes of each run were discarded to allow longitudinal magnetization to reach steady-state; differences in slice acquisition times were corrected using sinc interpolation; measurements of the static magnetic field (*B*_0_) performed in each scan session (except datasets 16, 17, 20, 21) were used to correct volumes for spatial distortion; and motion correction was performed using SPM5 (http://www.fil.ion.ucl.ac.uk/spm/). In general, we recommend that pre-processing include, at a minimum, discarding of the initial few volumes (if not already discarded by the scanner) and performing corrections for slice timing and motion.

### GLMdenoise model

**Figure 1:**
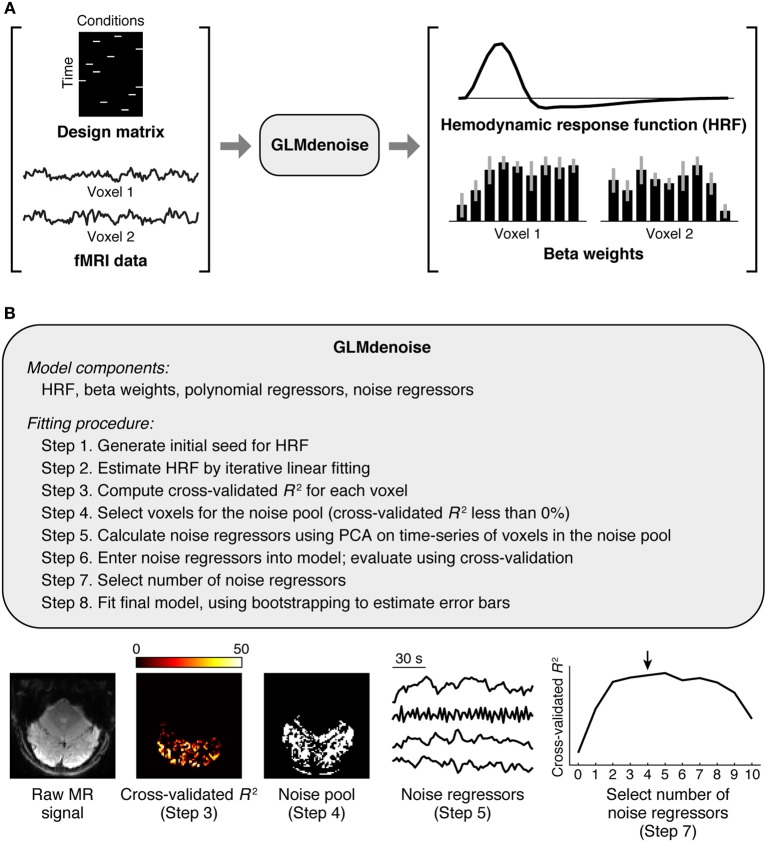
**Schematic of GLMdenoise**. **(A)** Inputs and outputs. GLMdenoise takes as input a design matrix (where each column indicates the onsets of a given condition) and fMRI time-series, and returns as output an estimate of the hemodynamic response function (HRF) and BOLD response amplitudes (beta weights). **(B)** Fitting procedure. The procedure consists of selecting voxels that are unrelated to the experiment (cross-validated *R*^2^ less than 0%), performing principal components analysis (PCA) on the time-series of these voxels to derive noise regressors, and using cross-validation to determine the number of regressors to enter into the model.

The GLMdenoise model consists of several components (Figure 1): an HRF characterizing the shape of the timecourse of the BOLD response, beta weights specifying the amplitude of the BOLD response to each condition, polynomial regressors characterizing the baseline signal level (which typically drifts) in each run (Liu et al., 2001; Kay et al., 2008a), and noise regressors capturing widespread BOLD fluctuations unrelated to the experiment (Behzadi et al., 2007; De Zwart et al., 2008; Bianciardi et al., 2009b). Some model components (HRF and beta weights) describe effects related to the experiment, while other model components (polynomial and noise regressors) describe effects unrelated to the experiment. The number of polynomial regressors included in the model is set by a simple heuristic: for each run, we include polynomials of degrees 0 through round(*L*/2) where *L* is the duration in minutes of the run (thus, higher degree polynomials are used for longer runs).

Formally, let *t* be the number of time points in a run, *r* be the number of runs, *c* be the number of conditions, *l* be the number of time points in the HRF, *p* be the number of polynomial regressors per run, and *g* be the number of noise regressors per run. The time-series data for a voxel are modeled as

y = (X * k) h + Pu + Gv + n

where **y** is a data vector (*tr* × 1), **X** is a design matrix where each column consists of zeros except for ones indicating the onsets of a given condition (*tr* × c), **k** is a vector with the HRF (*l* × 1), ^*^ denotes column convolution, **h** is a vector of beta weights (*c* × 1), **P** is a matrix whose columns are polynomial regressors (*tr* × *pr*), **u** is a vector of weights on the polynomial regressors (*pr* × 1), **G** is a matrix whose columns are noise regressors (*tr* × *gr*), **v** is a vector of weights on the noise regressors (*gr* × 1), and the vector **n** is a noise term (*tr* × 1). The component of the data related to the experiment (signal) is given by (**X** ^*^ **k**)**h**, while the component of the data unrelated to the experiment (noise) is given by **Pu** + **Gv** + **n**. (Note that the assumption that each run contains the same number of time points is only for notational convenience and is not essential. Also, since there are temporal breaks between runs, the convolution operation in the actual implementation does not extend across successive runs).

A few notes regarding the setup of the GLMdenoise model. First, data from multiple runs are analyzed in ensemble. Thus, a single beta weight is obtained for a condition that occurs across multiple runs. Second, each run obtains its own set of noise regressors. Third, the same HRF and the same set of polynomial and noise regressors are used for all voxels within a given dataset. Each voxel, however, obtains its own set of beta weights and its own weights on the polynomial and noise regressors. Finally, due to the use of cross-validation across runs (see section Quantification of accuracy), GLMdenoise requires at least two runs of data and assumes that conditions are repeated across runs. If only one run of data is collected, the run must be split into multiple segments before using GLMdenoise. We note, however, that there may be practical limitations to this splitting strategy. For example, because a separate set of noise regressors is constructed for each run, if the number of volumes in each run becomes too small, the inclusion of noise regressors may quickly result in overfitting. The code implementing GLMdenoise (see section Code) provides diagnostic figures that make it easy to assess the efficacy of GLMdenoise on any particular data preparation.

### Quantification of accuracy

Accuracy is quantified at several points in the fitting of the GLMdenoise model (see section Fitting procedure) as well as in the validation of the model [see section The Denoise Benchmark (DNB)]. To quantify accuracy, we use a cross-validation strategy in which a model is fit to a subset of the runs and the fitted model is used to predict the left-out runs. The model predictions include components of the data related to the experiment (HRF and beta weights) but do not include components of the data unrelated to the experiment (polynomial and noise regressors). The reason that these latter components are omitted is that the effects of these components cannot be predicted. (Signal drift, captured by polynomials, varies from run to run, and we do not expect the same signal drift to occur in different runs. As for the effects of the noise regressors, these effects can only be determined by peeking at the left-out data). Because of cross-validation and because the accuracy metric includes only experiment-related components, the accuracy values obtained through this procedure are lower than those that would be obtained in a typical GLM analysis.

The goodness-of-fit between model predictions and data is quantified using the coefficient of determination (*R*^2^):

$$R^{2}=100\times \left(1-\frac{\sum_{i}{\left(d_{i}-m_{i}\right)}^{2}}{\sum_{i}{\left(d_{i}-\bar{d}\right)}^{2}}\right)$$

where *d*_*i*_ indicates the *i*th data point in the measured time-series, *m*_*i*_ indicates the *i*th data point in the predicted time-series, and *d* indicates the mean of the measured time-series. The *R*^2^ value indicates the percentage of variance in the data predicted by the model, and has a maximum value of 100%. To ensure that models are not unduly penalized by failing to predict signal drift, the polynomial regressors are projected out from both the predicted time-series and the measured time-series before computing *R*^2^. Formally, let **J** be the projection matrix (**I** − **P**(**P**^T^**P**)^−1^**P**^T^) where **I** is the identity matrix (*tr* × *tr*). Then, *R*^2^ is calculated between the predicted time-series **J**[(**X** * **k**)**h**] and the measured time-series **Jy**.

Note that *R*^2^ is not the same as *r*^2^ (the square of Pearson's correlation coefficient) because *r*^2^ implicitly fits offset and gain parameters whereas *R*^2^ does not. Also, note that cross-validated *R*^2^ values can be less than 0%; this simply reflects the fact that a model can perform poorly predicting unseen data (*m*_*i*_ and *d*_*i*_ can diverge without limit). In several instances voxels are thresholded at an *R*^2^ cutoff of 0%; this is natural since 0% corresponds to the accuracy of a model that predicts no evoked BOLD responses (all beta weights equal to 0).

### Fitting procedure

The following is a description of the steps involved in applying GLMdenoise to a given dataset. Note that the steps are applied in sequence (there is no nesting of steps) and that each step involves all of the runs that are made available to GLMdenoise. In a later section [section The Denoise Benchmark (DNB)] we describe a testing framework in which runs are held out before calling GLMdenoise, thereby isolating GLMdenoise from the held-out data.

#### Step 1. Generate initial seed for HRF

We start by generating an initial seed for the HRF. The HRF represents the timecourse of the BOLD response and is assumed to be the same, up to a scale factor, for each condition. To generate the initial seed, we take a canonical HRF representing the response to a brief (0.1 s) stimulus and convolve this HRF with the appropriate square-wave function to predict the response for the condition duration used in the experiment.

The canonical HRF used in GLMdenoise was determined by fitting the double-gamma function implemented in SPM to experimental measurements of the HRF, and can be obtained using the following line of code: [0; spm_hrf(0.1,[6.68 14.66 1.82 3.15 3.08 0 48.9])] (note that the first time point corresponds to stimulus onset).

#### Step 2. Estimate HRF by iterative linear fitting

Using the HRF determined in Step 1 as a starting point, we estimate the optimal HRF (i.e., the HRF that best fits the data). This is accomplished using an iterative fitting strategy (Kay et al., 2008b): first, the HRF is fixed, and beta weights and polynomial weights are estimated using ordinary least-squares (OLS). Then, the beta weights are fixed, and the HRF and polynomial weights are re-estimated using OLS (the HRF is modeled using a fully flexible finite impulse response basis—see Dale, 1999). Next, the HRF is fixed, and beta weights and polynomial weights are re-estimated using OLS. This procedure is repeated until convergence of parameter estimates (defined as when the *R*^2^ between the previous and current HRF estimate is greater than 99%). After convergence, a *post-hoc* scaling of the HRF and beta weights is applied such that the peak value in the HRF is 1.

At each step where beta weights are estimated, the 50 best-fit voxels are determined, and these voxels are the ones fit in the subsequent step where the HRF is estimated. Excluding poorly-fit voxels in the HRF-estimation step is essential for obtaining good HRF estimates. In datasets with extremely low SNR, poor HRF estimates might be obtained. To compensate for such cases, we adopt the heuristic that if the *R*^2^ between the initial HRF and the fitted HRF is less than 50%, we simply use the initial HRF as the HRF estimate.

As an alternative to estimating the HRF, GLMdenoise can also accept a fixed, pre-determined HRF as an input. An implicit assumption of GLMdenoise is that a single HRF can describe the responses of all voxels in a dataset. However, the HRF may vary in shape across brain regions (Handwerker et al., 2012). It is possible to adapt GLMdenoise to account for HRF variations, but this is outside of the scope of the present paper.

#### Step 3. Compute cross-validated *R*^2^ for each voxel

Now that the HRF has been determined, we quantify accuracy of the GLM using leave-one-run-out cross-validation. This involves fitting the model to all runs except one, using the fitted model to predict the left-out run, and repeating this process for each run. Model predictions are concatenated across the left-out runs and then compared to the data using *R*^2^ (details provided in section Quantification of accuracy).

#### Step 4. Select voxels for the noise pool

The noise pool consists of voxels that are used to derive noise regressors. We select voxels for the noise pool according to two criteria. First, the cross-validated *R*^2^ value determined in Step 3 must be less than 0%. This criterion helps prevent voxels related to the experiment from entering the noise pool. Second, the mean signal intensity must be above a minimum threshold (specifically, one-half of the 99th percentile of mean signal intensity values across voxels). This criterion helps prevent voxels outside the brain from entering the noise pool. We could also accomplish this goal using an actual brain mask; we prefer intensity-based thresholding as it is simple and robust.

#### Step 5. Calculate noise regressors using PCA on time-series of voxels in the noise pool

For each run, we extract the time-series of the voxels in the noise pool, project out the polynomial regressors from each time-series, normalize each time-series to unit length, and perform principal components analysis (PCA) (Behzadi et al., 2007; Bianciardi et al., 2009b). The resulting principal components constitute candidate noise regressors.

#### Step 6. Enter noise regressors into model; evaluate using cross-validation

We refit the model to the data, systematically varying the number of noise regressors included in the model. For example, for two noise regressors, the model includes two additional regressors for each run, specifically, the two principal components that account for the maximum variance in the time-series of the voxels in the noise pool. Fitting is performed using leave-one-run-out cross-validation (as in Step 3). This produces cross-validated *R*^2^ values for each number of noise regressors.

Note that the noise regressors will, in general, have some correlation with the task-related regressors in the model. Indeed, the only way that beta weight estimates will change (thereby producing changes in cross-validation performance) is if there is some correlation between the noise and task regressors. By entering noise regressors into the model, variance in the data that was previously erroneously attributed to task regressors should now be captured by the noise regressors and lead to better beta weight estimates.

#### Step 7. Select number of noise regressors

**Figure 2:**
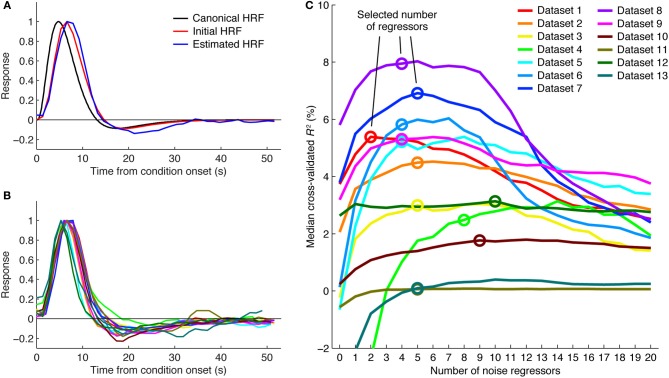
**Details of the GLMdenoise fitting procedure**. **(A)** HRF fitting. A canonical HRF representing the response to a brief stimulus (black curve) is convolved with the appropriate square-wave function to predict the response for the condition duration used in a given experiment (red curve). This is the initial seed for the HRF. Iterative linear fitting is then used to estimate the optimal HRF (blue curve). Results are shown for dataset 1 (curves are normalized to peak at one). **(B)** HRF estimates. Shown are HRF estimates obtained in different datasets. Color scheme same as in **(C)**. **(C)** Selecting the number of noise regressors. Voxels passing a minimum threshold are identified (voxels with cross-validated *R*^2^ greater than 0% under any number of noise regressors), and median cross-validated *R*^2^ values are calculated. The minimum number of regressors necessary to achieve within 5% of the maximum performance is selected. The performance curves are generally U-shaped, indicating that noise regressors help but too many noise regressors hurt performance.

To select the number of regressors to use, we first identify voxels that are likely to be related to the experiment. This is done by selecting all voxels that achieve a cross-validated *R*^2^ greater than 0% under any of the numbers of noise regressors. We then compute the median cross-validated *R*^2^ across these voxels for each number of regressors. We select the minimum number of regressors necessary to achieve a performance improvement that is within 5% of the maximum performance improvement (see Figure 2C). This slightly conservative selection strategy avoids potential overfitting and reduces susceptibility to random performance fluctuations. For example, a performance curve might generally peak at around 4 noise regressors but due to chance have a spike in performance at 10 noise regressors. The strategy also avoids unnecessary use of noise regressors in cases where performance curves have relatively flat plateaus (e.g., see dataset 11 in Figure 2C).

#### Step 8. Fit final model, using bootstrapping to estimate error bars

We perform a final fit of the model using the number of noise regressors determined in Step 7. To obtain error bars, we bootstrap the model 100 times. In each iteration, the model is fit to a bootstrap sample drawn from all available runs (e.g., if there are 10 runs, the model is fit to 10 runs drawn with replacement from the runs). The median across bootstrap results is used as the final model estimate, and one-half of the 68% range of bootstrap results is used as the estimate of standard error. (The idea behind the bootstrap is to use the data themselves as an estimate of the underlying population distribution and to use resampling to estimate confidence intervals). Finally, beta weights are converted to units of percent BOLD signal change by dividing by the mean signal intensity in each voxel.

The bootstrapping procedure described here occurs at the end of the GLMdenoise procedure (after the selection of the noise pool, the selection of the number of noise regressors, etc.). In contrast, a stricter bootstrapping procedure would resample the data prior to the GLMdenoise procedure (such that the entire GLMdenoise procedure is applied to each bootstrap sample). It is possible that because of the late application of bootstrapping, estimates of standard error provided by GLMdenoise may be somewhat optimistic (too small). One strategy for addressing this issue is to split the data prior to the application of GLMdenoise. For example, GLMdenoise could be independently applied to two halves of a given dataset (e.g., odd runs, even runs). Such an approach may be especially useful for classification-based studies that require strict separation between training and testing data. Finally, note that depending on the analysis workflow in which GLMdenoise is embedded, estimates of standard error may not be necessary. For example, if beta weight estimates are intended to be used in a second-level analysis, the bootstrapping procedure can be simply omitted from GLMdenoise.

### Code

MATLAB code implementing GLMdenoise is available at http://kendrickkay.net/GLMdenoise/. The code consists of standard (uncompiled) MATLAB functions, takes advantage of MATLAB's built-in multithreading, and requires only the Statistics Toolbox. To give a sense of the computational requirements of the code, we report here results for an example dataset: we ran GLMdenoise on dataset 10, which involved 35 conditions and a data dimensionality of 64 voxels × 64 voxels × 22 slices = 90,112 voxels and 10 runs × 265 volumes = 2,650 time points. The code was run on an Intel Xeon E5520 2.27 Ghz (8-core) workstation with 24 GB of RAM, a 64-bit Linux operating system, and MATLAB 7.9 (R2009b). Default parameters were used, including evaluating up to 20 noise regressors and performing 100 bootstraps of the final model. The data were loaded in single-precision format, resulting in a base memory usage of 1.0 GB of RAM. Code execution (including figure generation but excluding loading of the data from disk and saving the results to disk) took 10.4 min. The maximum memory usage over the course of code execution was 6.4 GB of RAM.

### Parameters

The main parameters of GLMdenoise are as follows: (1) The number of polynomial regressors included in the model. The default is to include polynomials of degrees 0 through round(*L*/2) for each run where *L* is the duration in minutes of the run. (2) The number of voxels considered when fitting the HRF. The default is 50. (3) The maximum number of principal components to evaluate. The default is 20, which is typically sufficient to cover the peaks of the cross-validation curves obtained in our datasets (see Figure 2C). (4) The number of bootstraps to perform for the final model fit. The default is 100, which provides reasonably accurate results with modest computational time and memory requirements. The default parameter values described here were used for all of the datasets in this paper. However, parameter values can be adjusted by the user if desired.

### The denoise benchmark (DNB)

The DNB (http://kendrickkay.net/DNB/) is a public database and architecture for evaluating denoising methods for task-based fMRI. The DNB consists of the 21 datasets described in this paper, a code framework that enables automatic evaluation of a candidate denoising method, and implementations of several different denoising methods (detailed in section Denoising methods). We used the DNB to compare the performance of GLMdenoise to that of other denoising methods.

The DNB is designed such that different denoising methods all conform to the same API. In essence, the API specifies that a denoising method should accept as input an fMRI dataset (a set of 3D volumes over time and a design matrix) and should return as output an estimate of task-related responses. Any denoising method that conforms to this API can be automatically evaluated by the DNB. The DNB allows direct comparison of different methods on the same datasets, and thus provides a means to adjudicate between methods. The DNB framework bears some similarity to the NPAIRS (non-parametric prediction, activation, influence and reproducibility resampling) framework (see Strother et al., 2002; Laconte et al., 2003; Churchill et al., 2012).

**Figure 3:**
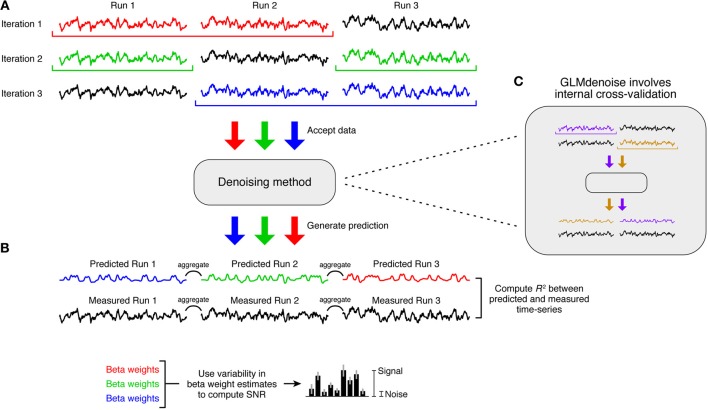
**The Denoise Benchmark (DNB)**. We designed an architecture that enables automatic evaluation of a candidate denoising method. **(A)** Cross-validation accuracy. Leave-one-run-out cross-validation is used to quantify the accuracy of the denoising method. In each iteration of this procedure, the denoising method is trained on all runs except one and is asked to predict the task-related signal in the left-out run. Predictions are aggregated across the left-out runs, and the accuracy of the predictions is quantified using coefficient of determination (*R*^2^). **(B)** Signal-to-noise ratio (SNR). Variability of beta weight estimates across the cross-validation iterations is used to estimate SNR. **(C)** Candidate denoising methods. Any denoising method that conforms to the prescribed application programming interface (API) can be evaluated in the DNB architecture. Note that the cross-validation used in the DNB is distinct from any internal resampling scheme that might be used by a denoising method (such as the cross-validation used within GLMdenoise).

The primary performance metric in the DNB is cross-validation accuracy, whereby a denoising method is evaluated on how well estimated task-related responses predict held-out data. This is implemented through a leave-one-run-out cross-validation procedure (Figure 3A). First, we leave out one run from the dataset and apply a denoising method to the remaining runs. (In datasets where conditions are grouped into run sets, the resampling procedure involves leaving out run sets instead of individual runs). We then use the estimate of task-related responses to predict the left-out run. This process is repeated for each run, the predictions are aggregated across left-out runs, and the overall accuracy of the predictions is quantified using *R*^2^ (see section Quantification of accuracy). To make minimal assumptions regarding signal drift, polynomials only up to degree 1 (i.e., constant and linear terms) are projected out from the predicted and measured time-series before computing *R*^2^.

There are two important characteristics of this cross-validation framework. First, the held-out data that a denoising method attempts to predict are raw data that have undergone only minimal pre-processing (see section Data pre-processing). These data have not been denoised because applying a denoising method to these data would pre-suppose the validity of the denoising method. Second, when using the DNB to evaluate GLMdenoise, there are actually two levels of cross-validation involved: an outer cross-validation used to evaluate the accuracy of the entire GLMdenoise procedure (Figure 3A) and an inner cross-validation used within GLMdenoise (Figure 3C). In practice, when applying GLMdenoise to a dataset, only the inner cross-validation is performed.

**Figure 4:**
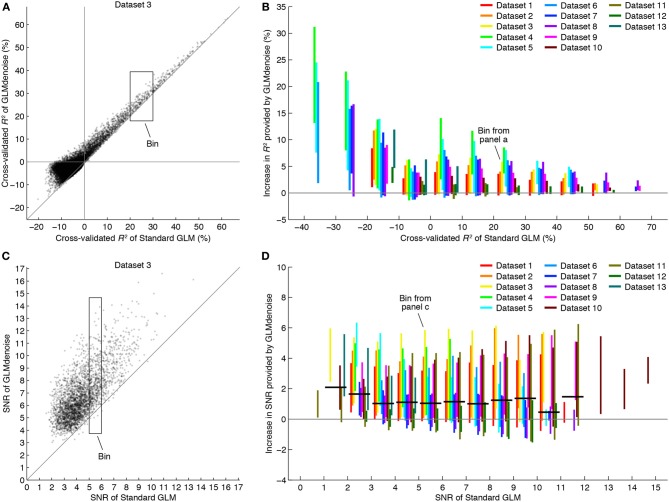
**GLMdenoise improves accuracy and reliability of BOLD response estimates**. Using the DNB, we compared the accuracy and reliability of GLMdenoise to that of an analysis involving no noise regressors (termed Standard GLM). **(A)** Comparison of *R*^2^ for an example dataset. Each dot indicates cross-validated *R*^2^ values for an individual voxel. **(B)** Summary of changes in *R*^2^. Voxels are binned according to the cross-validated *R*^2^ of Standard GLM (bin size 10%). For each bin with at least five voxels, we compute the increase in *R*^2^ provided by GLMdenoise and plot a line indicating the 95% range of results. GLMdenoise provides more accurate BOLD response estimates for nearly all voxels. **(C)** Comparison of SNR for an example dataset. Format same as **(A)**, except that only voxels passing a minimum threshold are shown (voxels with cross-validated *R*^2^ greater than 0% for either model). **(D)** Summary of changes in SNR. Format same as **(B)**, except that voxels are binned according to SNR (bin size 1). For each bin, we compute the median increase in SNR for each dataset and then the median of these values across datasets. The results are shown as thick black lines (for bins with contributions from at least two datasets). On average, GLMdenoise provides more reliable BOLD response estimates than Standard GLM.

The secondary performance metric in the DNB is SNR. To quantify SNR, we examine the stability of beta weight estimates across the cross-validation iterations (Figure 3B). The final beta weight estimate is computed as the mean across iterations, and the standard error is computed as the standard deviation across iterations, multiplied by the square-root of *n* − 1 where *n* is the number of iterations (this is the jackknife estimate of standard error—see Efron and Tibshirani, 1993). SNR is then computed by dividing the magnitude of the largest beta weight by the average standard error across beta weights. When comparing SNR across denoising methods, the magnitude of the largest beta weight is averaged across methods before computing SNR. This ensures that SNR differences across methods reflect only differences in the stability of beta weights, not differences in the magnitudes of beta weights (Kay et al., 2008a). We note that voxels with high SNR may tend to be those near large draining veins (Lee et al., 1995); in our reported results (Figure 4), voxels are separated according to SNR which allows one to inspect results for both low- and high-SNR voxels.

Although SNR is an intuitive metric that can be used to interpret the benefits of a denoising method, we emphasize that cross-validation accuracy takes ultimate precedence over SNR. The reason is that SNR does not measure the accuracy of a method but rather its reliability, and it is possible for a method to produce reliable but inaccurate results. For example, suppose one were to aggressively filter out low temporal frequencies in an fMRI dataset and then analyze the resulting data. Though the results may be quite stable across repeated measurements (high reliability), the results may very well be inaccurate since the filtering process will corrupt the portion of the task-related signal that resides within the filtered frequencies (Kay et al., 2008a). We therefore focus on the metric of cross-validation accuracy in this study.

We used the DNB to assess the cross-validation accuracy of a variety of denoising methods, and in Figure 6 we summarize performance by computing the median cross-validated *R*^2^ value achieved by each method on each dataset. The median is computed across voxels in each dataset that satisfy three conditions: (1) the voxel is contained within the brain mask calculated by FSL's Brain Extraction Tool (Smith, 2002), (2) the voxel has a cross-validated *R*^2^ value greater than 0% under at least one of the denoising methods being compared, and (3) the voxel satisfies condition 2 after slight spatial smoothing of the *R*^2^ volumes (3D Gaussian filter, FWHM equal to 1.5 times the voxel size in each dimension). The purpose of conditions 1 and 3 is to ignore random speckles in the *R*^2^ volumes (false positives) and to help focus the performance summary on brain regions with task-related signals. Comprehensive results showing all voxels in all datasets are available at the DNB web site.

### Denoising methods

In this section we describe the denoising methods that we tested using the DNB. To ensure that methods differed only in their beta weight estimates, the HRF estimate for a given dataset was fixed and used across all methods. Thus, differences in cross-validation performance can be directly attributed to differences in the quality of beta weight estimates.

#### Standard GLM

*Standard GLM* is identical to GLMdenoise except that no noise regressors are used. (Thus, only Steps 1, 2, and 8 of the GLMdenoise fitting procedure are used.) This method provides a measure of baseline performance.

#### Global signal

*Global signal* is identical to Standard GLM except that for each run, one additional nuisance regressor is used: a regressor that is computed by taking the mean of each functional volume (Desjardins et al., 2001).

#### Motion regressors

*Motion regressors* is identical to Standard GLM except that motion parameter estimates from the SPM5 motion correction procedure are included as additional nuisance regressors (Friston et al., 1996; Johnstone et al., 2006). There are six additional regressors for each run (corresponding to three translation parameters and three rotation parameters).

#### ICA-based denoising

We designed a denoising procedure based on FSL's MELODIC (FSL 5.0, http://fsl.fmrib.ox.ac.uk/fsl/fslwiki/MELODIC), a utility that implements independent components analysis (ICA) of fMRI data (Mckeown et al., 2003; Jenkinson et al., 2012). For our purposes, it is essential that an ICA-based denoising procedure automatically determines which components are signal (task-related) and which are noise (task-unrelated). This is not only to avoid subjectivity and to promote reproducibility, but also for practical reasons, as there are many runs and datasets in the DNB. Moreover, given the event-related designs used in the datasets, it may be quite difficult to tell by inspection whether a given component represents signal or noise.

The ICA-based denoising procedure is as follows. (1) We detrend the data by projecting out polynomials from each run (the maximum polynomial degree is the same as used in GLMdenoise). However, the mean of each run is preserved. (2) For each run, we run MELODIC, which produces a set of component timecourses. (3) For each timecourse, we calculate the amount of variance explained by the task-related regressors corresponding to that run. For comparison, we repeat this calculation for 1,000 randomly shuffled versions of the timecourse. (4) If the amount of variance explained in the actual timecourse is more than *c* standard deviations away from the mean of the shuffled results (in the positive direction), the timecourse is marked as signal. Otherwise, the timecourse is marked as noise. (5) After processing all component timecourses, we use FSL's *fsl_regfilt* utility to remove the identified noise components from the data. (6) The denoised data are then analyzed using Standard GLM.

We evaluated two variants of the ICA-based denoising procedure. *ICA (conservative)* is the procedure using a threshold of *c* = 0. This is a conservative threshold that tends to retain components as signal (i.e., it is cautious about throwing away useful signals). *ICA (liberal)* is the procedure using a threshold of *c* = 3. This is a liberal threshold that aggressively identifies components as noise (i.e., it is less cautious about throwing away useful signals).

#### GLMdenoise

*GLMdenoise* is the novel denoising method described in this manuscript.

*GLMdenoise (scrambled)* is identical to GLMdenoise except that the phase spectrum of each noise regressor is scrambled before noise regressors are entered into the model. This manipulation serves as a control: presumably, the precise temporal information contained in the noise regressors is critical for accurate characterization of noise in the time-series data. Phase-scrambled noise regressors should therefore fail to provide substantial denoising benefits.

*GLMdenoise (no exclusion)* is identical to GLMdenoise except that there is no exclusion of voxels with task-related signals from the noise pool (specifically, in Step 4 of the GLMdenoise fitting procedure, the exclusion of voxels based on cross-validated *R*^2^ is omitted). This manipulation provides insight into whether the exclusion of task-related signals matters. In theory, if substantial task-related signals are present in the noise pool, the noise regressors that are derived will contain a mixture of both signal and noise and will therefore be less effective at denoising the task-related estimates.

#### RETROICOR/RVHRCOR

Datasets 14–21 of the DNB involved collection of physiological data, which enables the use of the RETROICOR/RVHRCOR denoising techniques (Glover et al., 2000; Chang et al., 2009). Because the physiological regressors computed in these techniques are sensitive to slice timing, we apply the techniques as part of the data pre-processing stream. The procedure is as follows: (1) RETROICOR and RVHRCOR regressors are computed from the physiological data (code available at https://github.com/cni/nims/blob/master/nimsdata/nimsphysio.py). (2) The physiological regressors are orthogonalized with respect to polynomials of up to degree 2 (constant, linear, quadratic). (3) The first five volumes of each run are discarded. (4) The physiological regressors and polynomials are fit to the time-series data from each voxel. (5) The component of the fit that is attributed to the physiological regressors is subtracted from the data. (6) The data undergo the remaining pre-processing steps (slice time correction, spatial undistortion, motion correction). (7) Finally, the data are analyzed using Standard GLM.

We evaluated two versions of this denoising procedure. *RETROICOR* involves removing only the RETROICOR regressors (a set of eight regressors that model the effects of cardiac pulsation and respiratory motion). *RETROICOR* + *RVHRCOR* involves removing both the RETROICOR regressors and the RVHRCOR regressors (a set of four regressors that model the effects of heart rate and respiratory variations).

#### Omnibus

*Omnibus* is a denoising method that combines several of the methods described above. If physiological data are available, the method involves removing the RETROICOR and RVHRCOR regressors (see section RETROICOR/RVHRCOR) and then analyzing the data using Standard GLM, including global signal (see section Global signal) and motion parameters (see section Motion regressors) as nuisance regressors. If physiological data are not available, the method involves analyzing the data using Standard GLM, including global signal and motion parameters as nuisance regressors.

## Results

### Basic mechanics of GLMdenoise

GLMdenoise is a variant of the GLM that is commonly used in fMRI analysis (Dale, 1999; Monti, 2011; Poline and Brett, 2012). The GLMdenoise model consists of an HRF and beta weights, which describe effects related to the experiment and are of primary interest, as well as polynomial and noise regressors, which describe nuisance effects (Figure 1A). To determine the noise regressors, an initial model without noise regressors is used to identify voxels unrelated to the experimental paradigm (the noise pool) and PCA is performed on the time-series of these voxels (Figure 1B). The basic idea of using PCA to derive noise regressors has been presented previously (Behzadi et al., 2007; Bianciardi et al., 2009b).

Two aspects of the GLMdenoise fitting procedure are illustrated for further clarification. One is that GLMdenoise includes estimation of the HRF from the data (Figures 2A,B). This is useful since the HRF may vary across subjects (Handwerker et al., 2012). Another aspect is that cross-validation is used to determine the appropriate number of noise regressors to include in the model. Including noise regressors typically improves cross-validation performance, indicating that it is beneficial to use regressors to account for noise in the data (Figure 2C). Notice, however, that in most cases, after adding a certain number of noise regressors, performance starts to decline. This indicates that using too complex of a model for the noise risks overfitting. Also notice that the optimal number of noise regressors varies across datasets. This highlights the importance of using a data-driven approach to set the number of noise regressors.

### Improvements in cross-validation and SNR

The consistency and size of the cross-validation improvements suggest that including noise regressors improves accuracy of GLM estimates. However, technically, the selection of the number of regressors and the selection of the noise pool are parameters of the fitting process, and these must be evaluated to obtain a strict test of accuracy.

To rigorously evaluate GLMdenoise, we designed an architecture that subjects the entire GLMdenoise method to a cross-validation procedure (thus, there are two levels of cross-validation involved). The architecture is termed the DNB (Figure 3). The architecture allows different analysis procedures to be tested, including other denoising methods (see section Comparison with other denoising methods). Here we focus on comparing the performance of GLMdenoise to that of an analysis that omits the noise regressors, termed Standard GLM.

Examining the cross-validation accuracy of GLMdenoise and Standard GLM, we find that GLMdenoise consistently improves accuracy, though the exact magnitude of the improvement varies across datasets (Figures 4A,B). Notice that improvements are found even for voxels for which Standard GLM produces a cross-validated *R*^2^ value less than 0%. Even though these voxels are initially deemed unrelated to the experiment and are used to construct the noise pool, the denoising process is still able to improve the GLM estimates for these voxels (in some cases producing cross-validated *R*^2^ values greater than 0%).

SNR is a metric whose units are easier to interpret than cross-validated *R*^2^ values. To better understand the magnitude of the improvements provided by GLMdenoise, we quantified SNR in beta weight estimates (Figure 3B). The results demonstrate that GLMdenoise substantially improves SNR compared to Standard GLM (Figures 4C,D). We find that the amount of SNR improvement is fairly constant across SNR levels. For example, a voxel with a base SNR of 4 under Standard GLM typically experiences an increase in SNR to 5 under GLMdenoise, just as a voxel with a base SNR of 8 typically experiences an increase in SNR to 9.

**Figure 5:**
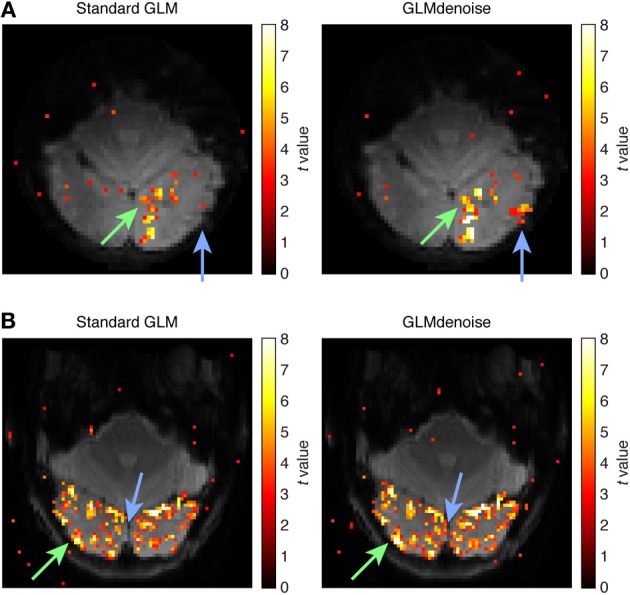
**Example activation maps**. As an intuitive way to visualize SNR improvements, we show maps of *t*-values obtained using Standard GLM and maps obtained using GLMdenoise. Maps have been thresholded at *t* > 3 and are overlaid on the mean functional volume. **(A)** Activation map for dataset 3, slice 11, condition 31. The green arrow indicates an activated region that exhibits substantial increases in *t*-values when using GLMdenoise. The blue arrow indicates a region that exhibits activation under GLMdenoise but not under Standard GLM. **(B)** Activation map for dataset 7, slice 11, condition 24. Format same as **(A)**.

Finally, the improvements in SNR provided by GLMdenoise can be visualized using activation maps (Figure 5). Notice that the maps produced by GLMdenoise have improved statistical power compared to those produced by Standard GLM. Of course, the depicted maps represent just a small subset of the full set of results (provided in Figure 4).

### Comparison with other denoising methods

We used the DNB to compare the performance of GLMdenoise to that of several popular denoising methods. To allow comparison with methods that require physiological data (e.g., RETROICOR/RVHRCOR), the DNB includes several datasets that were acquired with concurrent physiological monitoring (pulse oximeter and respiratory belt). The DNB is publicly available (including data and code implementations of the denoising methods), and we welcome efforts to implement and test other methods.

**Figure 6:**
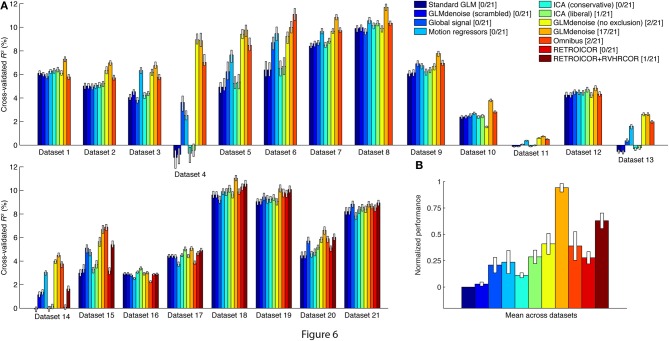
**GLMdenoise outperforms other denoising methods**. Using the DNB, we quantified the cross-validation accuracy of a variety of denoising methods on a large number of datasets. **(A)** Results for individual datasets. For each dataset, we summarize the performance of a method by plotting the median cross-validated *R*^2^ value obtained under that method. Error bars indicate 68% confidence intervals and were obtained via bootstrapping. **(B)** Overall results. To summarize performance across datasets, we normalize the pattern of results from each dataset such that Standard GLM corresponds to 0 and the best-performing method corresponds to 1. We then compute the mean of this pattern across datasets (error bars indicate standard error of the mean). As an alternative performance summary, we count the number of datasets for which a given method achieves the best or nearly the best performance (specifically, the number of datasets for which the median performance of a method either is the best or provides at least 95% of the performance improvement provided by the best method). The number of datasets (out of 21 total datasets) is indicated in the legend.

A summary figure shows the median cross-validation accuracy of each denoising method on each dataset (Figure 6A). The datasets with physiological data are located in the bottom row. Cross-validation accuracy varies substantially across datasets, reflecting the fact that different experiments produce different levels of BOLD activations (i.e., some visual stimuli drive responses better than others). Within individual datasets, however, the pattern of performance across denoising methods is relatively consistent. To see this more clearly, we normalize the pattern of results found for each dataset and average across datasets (Figure 6B). The best-performing method is GLMdenoise.

The performance of the other denoising methods provides insight into the types of noise that GLMdenoise may remove. Global signal (a method that includes the mean of each functional volume as a nuisance regressor), Motion regressors (a method that includes six motion parameters as nuisance regressors), and RETROICOR/RVHRCOR (a method that includes nuisance regressors constructed from physiological data) all produce some improvement in cross-validation accuracy. This suggests that the noise removed by GLMdenoise may include global BOLD modulations, residual effects of head motion, and physiological noise. Interestingly, Omnibus (a method that combines the global, motion, and physiological regressors) often performs well but is inconsistent, sometimes performing even worse than Standard GLM (e.g., datasets 1, 16). This highlights the important point that more nuisance regressors is not always better: using too many nuisance regressors can result in overfitting and poor task estimates.

Finally, we tested a couple of variants of GLMdenoise as control cases. GLMdenoise (scrambled) is a variant of GLMdenoise where the phase spectra of the noise regressors are scrambled before entering the model. This method performs about as poorly as Standard GLM, confirming that the specific timecourses of the noise regressors are essential to achieve denoising. GLMdenoise (no exclusion) is a variant of GLMdenoise where all voxels are allowed to enter the noise pool, even if they have high cross-validated *R*^2^ values. This method performs substantially worse than GLMdenoise in many datasets (e.g., datasets 8, 18), demonstrating the importance of the exclusion step: the noise regressors will be most effective at accounting for noise if they do not also contain task-related signals in them. This is consistent with the finding that GLMdenoise (no exclusion) performs about as well as GLMdenoise in datasets where BOLD activations are weak (e.g., datasets 11, 13).

## Discussion

### Empirical and theoretical advantages of GLMdenoise

GLMdenoise is a fast, automated denoising technique for task-based fMRI that requires no extra data. GLMdenoise can be used to extract greater amounts of information from existing sets of data, reduce data collection time, or increase the number of conditions sampled in an experiment. We have shown that GLMdenoise outperforms a number of other denoising methods on a variety of datasets. To facilitate usage of this new tool, we make freely available MATLAB code implementing GLMdenoise. In addition, we provide the DNB, which facilitates direct, quantitative comparisons between denoising methods.

Besides superior empirical performance, there are several theoretical reasons to prefer GLMdenoise over other denoising methods. One is that the noise regressors derived in GLMdenoise are general and can encompass many different types of noise, including motion-related noise, physiological noise, neural noise, etc. Thus, GLMdenoise has the potential to correct for multiple noise sources. Second is that because noise regressors are derived from the data, GLMdenoise relies on minimal assumptions regarding the nature of the noise. For example, the denoising strategy of including motion parameters as regressors assumes that motion-related noise can be described as a linear function of the motion parameters. In contrast, GLMdenoise is agnostic with respect to how motion-related noise is manifested in the data (the noise can bear a complex non-linear relationship to motion parameters).

A third reason to prefer GLMdenoise is that it is self-calibrating. As our results have shown, the accuracy of task estimates can decrease if too many noise regressors are used (e.g., Omnibus on dataset 1 in Figure 6). Moreover, even a policy of using a small, fixed number of noise regressors does not guarantee good results (e.g., Motion regressors on dataset 17 in Figure 6). The reason for this variability in results is that the efficacy of including noise regressors depends on the magnitude of the noise, the magnitude of the task-related signals, and the amount of correlation between the noise and task-related signals, all of which may depend on the subject and the experiment. GLMdenoise addresses this issue by using cross-validation to determine the proper number of noise regressors to use on each given dataset. This adaptive, data-driven approach ensures that GLMdenoise consistently improves (or at least preserves) the quality of task estimates.

### Caveats and limitations

An implicit assumption in GLMdenoise is that all time-series modulations that correlate with experimental conditions are signals of interest. However, a problematic scenario is one in which head motion is reliably correlated with the experimental conditions. In such a scenario, we may find that a GLM fitted to the data may produce spurious task estimates that cross-validate well. The problem of task-correlated motion is not specifically addressed by GLMdenoise. In cases of task-correlated motion, independent metrics of head motion (e.g., motion parameters) may be valuable for disentangling motion-related effects from task-related effects.

The datasets described in this paper were collected from relatively well-behaved subjects and are free of gross imaging artifacts. To denoise datasets containing extreme head motion and/or substantial image artifacts, strategies beyond GLMdenoise may be necessary. In particular, ICA-based denoising approaches might prove valuable for these types of datasets. This may explain, for example, why ICA provided substantial improvements on one of our datasets (dataset 16). An interesting future direction is to incorporate datasets with severe artifacts into the DNB.

## Author contributions

Kendrick N. Kay conducted the experiments and analyzed the data. Ariel Rokem and Jonathan Winawer provided conceptual guidance. Ariel Rokem provided software and programming advice. Robert F. Dougherty aided in the collection and analysis of physiological data. Kendrick N. Kay and Brian A. Wandell wrote the manuscript. All authors discussed the results and edited the manuscript.

### Conflict of interest statement

The authors declare that the research was conducted in the absence of any commercial or financial relationships that could be construed as a potential conflict of interest.
